# Exploring antimicrobial stewardship education and training support for community pharmacists in the United Kingdom

**DOI:** 10.1080/20523211.2024.2365224

**Published:** 2024-06-28

**Authors:** Sandra J. Martin, Sarah Chadwick, Mina Bakhit, Kevin J. Frost, Gillian Hawksworth, Mamoon A. Aldeyab

**Affiliations:** a School of Pharmacy & Medical Sciences, University of Bradford, Bradford, UK; b NHS West Yorkshire Integrated Care Board, White Rose House, Wakefield, UK; c Institute for Evidence-Based Healthcare, Bond University, Gold Coast, Australia; d Pharmacy Department, Airedale NHS Foundation Trust, Keighley, UK; e Department of Pharmacy, School of Applied Sciences, University of Huddersfield, Huddersfield, UK

## Background

A key objective of both the World Health Organisation (WHO) Global Action Plan on Antimicrobial Resistance (AMR) (World Health Organisation [WHO], 2016) and the UK national action plan (Department of Health and Social Care et al, 2024) is to improve awareness and understanding through effective communication, education, and training (Cooke, 2022). Successful initiatives promoting Antimicrobial stewardship (AMS) in community healthcare organisations in England include national programmes (National Institute for Health and Care Excellence [NICE], 2015) and toolkits such as the Treat Antibiotics Responsibly, Guidance, Education, and Tools (TARGET) (Parekh et al., 2023b) and the Antibiotic Guardian campaign (Ashiru-Oredope et al., 2018; Seaton et al., 2022). In England, the Pharmacy Quality Scheme (PQS) incentivises AMS activity in community pharmacies. Since 2021/22, it has mandated the use of the TARGET antibiotic checklist, which supports pharmacist review of each prescribed antibiotic (Hayes et al., 2023).

The 2022/23 PQS enhanced this clinical role by providing tools that facilitate the implementation of shared decision-making in consultations for patients with respiratory and urinary tract infections before dispensing an antibiotic and promoting the use of TARGET Treating Your Infection (TYI) leaflets aimed at supporting appropriate antibiotic use (Parekh et al., 2023a). To guide antibiotic supplies for sore throats in Wales in 2018, pharmacists received additional training to supply patients with antibiotics in line with a patient group direction (PGD) (Mantzourani et al., 2019). The NHS England Pharmacy First scheme (2024) built on these services, enabling pharmacists to supply prescription-only medicines, including antimicrobials, to treat seven common health conditions without a General Practitioner (GP) consultation (NHS England [NHSE], 2023a).

As the community pharmacy contractual framework has developed with new and expanded services (NHSE, 2019, 2023b), healthcare teams have had to evolve and adapt to respond to the burden of antimicrobial resistance (AMR).

Clinical expertise of pharmacists must be fully utilised to ensure the appropriate use of antibiotics and reduce AMR. In a semi-structured focus group with UK community pharmacists, we explored what education and training is required to confidently contribute to prescribing decisions and patient counselling regarding antimicrobial use.

### Findings and key lessons learned

#### Pharmacy as a key provider of health advice on antimicrobials and antimicrobial stewardship

Pharmacists agreed they are most suited to advise patients about antibiotics, feeling confident in their ability to deliver effective antimicrobial advice. They highlighted their unique position and accessibility as key factors that make them effective agents for patient education on this topic. Pharmacists prioritise patient safety over external pressures when managing antibiotic prescriptions with decisions grounded in clinical judgment. They highlighted concerns about factors that may lead to overprescribing, including the mismatch between the GP-prescribed dose and packaging, leaving patients with leftover antibiotics. Variability in expertise within the team was also highlighted, particularly among counter staff who rely on self-development for their knowledge. This poses challenges in providing consistent advice and raising concerns about the quality of guidance from non-pharmacist staff. There was agreement on the importance of ongoing participation in pharmacy quality schemes for antimicrobial stewardship.

#### Challenges and concerns with Pharmacy First Initiative and the need for training

Pharmacists highlighted the significant challenges they faced while implementing the Pharmacy First initiative. Pharmacists expressed concerns about inadequate clinical training and lack of teaching or support when the initiative started, leaving them facing an overwhelming information load. Consequently, it became their burden to undertake self-development; subjectively judging which information to read, which in turn produced a sense of feeling unprepared.

#### Pharmacists’ role in the management of patients with acute infections

Pharmacists described their critical role in: (i) diagnosing the nature of infections including severity, appropriateness of antibiotic management and whether urgent care is needed; and in (ii) educating patients that their condition is minor and self-limiting, which could be managed simply with over the counter (OTC) medications.

#### Pharmacists are concerned about patients’ expectations for antibiotic prescriptions, including refills

Pharmacists in the focus group believed that patients were confused about the difference between bacterial and viral infections, influencing their expectations for antibiotic treatment. Patients referred to pharmacies often arrive with an assumption of receiving antibiotics, making it a challenge for them to consider alternatives. Another concern raised was related to antibiotics prescribed at walk-in-centres, with a lack of clarity regarding how they are dispensed, and antibiotics intended for ‘rescue bags,’ often deemed inappropriate by healthcare providers.

Pharmacists in the focus group highlighted that, despite pressures, they do not automatically refill prescriptions; instead, they focus on educating patients about the appropriate use of antibiotics, including the importance of following prescribed durations and seeking further evaluation from a GP if needed. However, this can be challenging, especially when patients are frustrated by persistent symptoms. Pharmacists highlighted the need for enhanced public education on antibiotic aftercare and treatment.

Pharmacists emphasised that improper patient referrals from GP clinics were another factor that often burdened them with additional responsibilities to rectify misunderstandings. Moreover, the three-day rule of antibiotic prescribing restricts management to patients who need earlier care. It was noted that patients might become adept at responding to screening questions in a manner that falsely meets the criteria for antibiotics. This belief that some patients would abuse the system was a major concern; yet pharmacists felt compelled to push Pharmacy First and prescribe antibiotics to meet targets even when not medically necessary. This pressure was perceived to arise from both internal management and external healthcare policies. It was noted that the obligation to maintain good relationships with patients often leads to unnecessary prescriptions to avoid difficult consultations or delays in treatment.

#### A patient-centred approach to healthcare advice emphasises the importance of personalising guidance based on individual needs

Pharmacists reported that their decision on the type of advice to provide was heavily influenced by each patient’s presenting symptoms, unique needs, and past experiences. Understanding a patient’s background with a condition or treatment was highlighted as essential to ensure that advice was not only relevant but also effective. Safety netting emerged as a critical component of the advice-giving process. Pharmacists emphasised the necessity of ensuring that patients understand their infection, including possible prognoses, actions to take under various scenarios, what to do next, and the importance of follow-up if conditions do not improve.

#### Pharmacy services – enhancing accessibility, building trust, and maximising healthcare value

Participants frequently emphasised the ease afforded by the no-appointment-necessary approach of pharmacies. This accessibility facilitates spontaneous consultations and enhances convenience for patients who might otherwise delay seeking care. Pharmacists highlighted that patients appreciate being able to receive advice promptly, without the long wait times typically associated with booking GP appointments. The simplicity of referrals from pharmacies to GPs was highlighted as an essential aspect of pharmacy services, streamlining the healthcare journey for patients and ensuring they receive effective care. Pharmacists noted a high level of patient trust, which fosters open and honest discussions, enabling pharmacists to address concerns and enhance comfort during consultations. The focus group participants reported that patients particularly valued the ability to provide immediate reassurance about health conditions; alleviating anxieties and clarifying treatment paths; thereby playing a crucial role in the immediate management of health issues. It is believed that by consulting pharmacists for minor health issues, patients feel they are helping to alleviate the burden on GPs under the strain of NHS constraints.

#### Pharmacists’ involvement in the use of diagnostic tests, particularly those that could be utilised at home

Pharmacists reported minimal participation in reviewing diagnostic tests within multidisciplinary teams. Pharmacists’ familiarity with interpreting results is primarily gained through experience with patient cases over time. Whilst the use of lateral flow tests during the COVID pandemic was widespread, similar devices in infectious diseases are limited. Other conditions have simple at-home monitoring tests like blood glucose or cholesterol tests. Pharmacists are ideally placed to support greater use of infectious disease tests at home.

## Conclusion

This work provides insight into community pharmacists providing AMS through reviewing antibiotic prescriptions, advising patients with minor infections, supplying antimicrobials through patient group directions and raising public awareness of AMR. The pharmacists felt confident in reviewing antibiotic prescriptions and delivering effective antimicrobial advice to patients. Building rapport with patients when assessing infection, providing valuable services for managing minor infections for patients, liaising with members of the multidisciplinary team, and supporting local NHS services promoted positive feelings of worth. Opportunities for promoting AMS through public health messaging and supporting safe, effective prescribing were considered key roles. Challenges included patient pressure for supplying antimicrobials, particularly with the new Pharmacy First initiative, and consistency of staff training on AMS. Pharmacists would have liked more ‘point of care’ diagnostics to support the diagnosis of infections. Of note, the timing of the conducted focus group was soon after the launch of the Pharmacy First scheme. Future work should include further focus groups to increase the community pharmacist voice and at a later time so that the Pharmacy First scheme has become more established.
